# Putative Allele of *D10* Gene Alters Rice Tiller Response to Nitrogen

**DOI:** 10.3390/plants13233349

**Published:** 2024-11-29

**Authors:** Tamanna Islam Rimi, Meirong Zhang, Ruixin Zhang, Zhe Zhang, Xueyu Leng, Jiafang Han, Sihan Meng, Wen Du, Zhongchen Zhang

**Affiliations:** 1 College of Agriculture, Northeast Agricultural University, 600 Changjiang Road, Harbin 150030, China; tamannaislam2209@yahoo.com (T.I.R.); 18846914861@163.com (M.Z.); 19917625056@163.com (R.Z.); jiudragon1215@163.com (Z.Z.); 17561746702@163.com (X.L.); hanjiafang0@163.com (J.H.); msh528172023@163.com (S.M.); 18245666208@163.com (W.D.); 2 National Key Laboratory of Smart Farm Technologies and Systems, Harbin 150030, China

**Keywords:** rice (*Oryza sativa* L.), dwarf and multiple tillering, *P47DT1*, nitrogen response

## Abstract

The number of tillers in rice significantly affects final yield, making it a key trait for breeding and nitrogen-efficient cultivation. By investigating agronomic characteristics, we analyzed phenotypic differences between the wild-type P47-1 and the mutant *p47dt1*, performing genetic analysis and gene mapping through population construction and BSA sequencing. The *p47dt1* mutant, exhibiting dwarfism and multiple tillering, is controlled by a single gene, *P47DT1*, which is tightly linked to *D10*. A single base mutation (T to G) on chromosome 1 alters methionine to arginine, supporting *D10* as the candidate gene for *p47dt1*. To investigate nitrogen response in tillering, KY131 (nitrogen-inefficient) and KY131*^OsTCP19^*^-H^ (nitrogen-efficient) materials differing in *TCP19* expression levels were analyzed. Promoter analysis of *D10* identified TCP19 as a nitrogen-responsive transcription factor, suggesting *D10*’s potential role in a TCP19-mediated nitrogen response pathway. Further analysis of P47-1, p47dt1, KY131, and KY131*^OsTCP19^*^-H^ under different nitrogen concentrations revealed *p47dt1*’s distinct tiller response to nitrogen, altered nitrogen content in stems and leaves, and changes in *TCP19* expression. Additionally, *D10* and *TCP19* expression levels were lower in KY131*^OsTCP19^*^-H^ than KY131 under identical conditions. In summary, *P47DT1*/*D10* appears to modulate nitrogen response and distribution in rice, affecting tiller response, possibly under TCP19’s regulatory influence.

## 1. Introduction

Rice (*Oryza sativa* L.) is one of the world’s most important staple crops, providing food for over half of the global population [1]. In China, rice is a primary grain crop, and its production is closely tied to the nation’s food security. The number of tillers, which are side shoots that grow from the base of the rice plant, is a key determinant of rice yield. Each tiller can produce a panicle, which directly contributes to grain production. Optimizing the tiller number is a critical objective in rice breeding programs to enhance yield potential [2,3]. As one of the essential nutrient elements in plant growth and development, nitrogen is very important for the growth of rice, especially the growth of rice tillers [4]. Therefore, studying the genes related to nitrogen response in rice is crucial for improving rice yield.

The conditions affecting rice tillering are complex, involving both endogenous hormones and external environmental factors [5]. Plant hormones, such as gibberellins (GAs), cytokinins (CKs), brassinosteroids (BRs), auxins, and strigolactones (SLs), regulate plant structure in rice [6]. Current research indicates that cytokinin, auxin, and strigolactone are the main factors affecting rice tillering [7,8,9,10,11]. Cytokinins promote tiller initiation by stimulating the outgrowth of axillary buds, while auxins, which are transported downward from the shoot apex, suppress tillering by maintaining apical dominance, preventing axillary bud growth. Strigolactones, discovered as a new class of plant hormones in 2008, play an inhibitory role in tiller development by suppressing the outgrowth of axillary buds [12,13]. Mutations in SL biosynthesis or signaling can lead to plant dwarfing and an increased tiller number [6].

Several key genes involved in the strigolactone synthesis pathway have been identified in rice. For example, the *D17*, *D10*, and *D27* genes encode enzymes that are critical for SL biosynthesis. *D17* encodes OsCCD7, which inhibits tiller growth by limiting lateral branching, thereby negatively regulating tillering [14,15]. Similarly, *D10*, encoding OsCCD8, functions in the carotenoid cleavage pathway [16], and its expression is induced by auxin, which can reduce auxin transport capacity and promote cytokinin synthesis [17,18]. *D27*, a newly identified gene in the strigolactone synthesis pathway, enhances auxin transport polarity when mutated, leading to an increased tiller number and a dwarfing multi-tillering phenotype [19,20].

In addition to these hormonal controls, brassinosteroids (BRs), a class of polyhydroxy steroids, also regulate rice tillering. BR signaling is mediated by the DLT transcription factor (involved in brassinosteroid (BR) signaling, essential for regulating cell growth and tillering), which promotes cell division and elongation, impacting tiller formation [21,22]. The interaction between nitrogen availability and BR signaling is evident through the regulation of OsTCP19, a transcription factor that combines with the DLT promoter to control brassinosteroid synthesis and tiller development [23]. Understanding how nitrogen interacts with BRs is crucial for unraveling the hormonal regulation of rice tillering. 

The main environmental factors affecting rice tillering include temperature, light, water, and nutrients [24]. However, in actual field production, temperature, light, water, and other factors are usually stable and are not factors that can cause significant changes in the tillering of rice. In general, producers can directly and effectively regulate nutrient levels only, especially nitrogen levels. Nitrogen affects plant growth and development mainly through the nutrient level and signal transduction pathway, and there is a cooperative regulation relationship between these two aspects [25]. It has been shown to decrease strigolactone content in rice nodes, indicating that nitrogen may promote tillering by inhibiting the SL pathway. This nitrogen–strigolactone interaction is further supported by the finding that nitrogen application increases cytokinin levels, promoting tiller formation [26]. Moreover, in strigolactone-deficient mutants, such as *d3* and *d10*, nitrogen content in the leaves is reduced compared to wild-type plants. Treatment with GR24 (a synthetic strigolactone analog used to study SL signaling and its effects on plant development) restores normal nitrogen content in these mutants, highlighting the importance of strigolactones in coordinating nitrogen distribution and tillering [27,28].

Despite these advances, the molecular mechanisms through which nitrogen and plant hormones interact to regulate tillering remain largely unknown. This study aims to explore the relationship between nitrogen, plant hormones, and rice tillering by focusing on the dwarf and multi-tillering mutant *p47dt1*, thereby contributing to a better understanding of how nitrogen-responsive genes influence rice tillering.

## 2. Results

### 2.1. p47dt1 Mutant Showed Dwarfing Multiple Tillers

**Figure 1:**
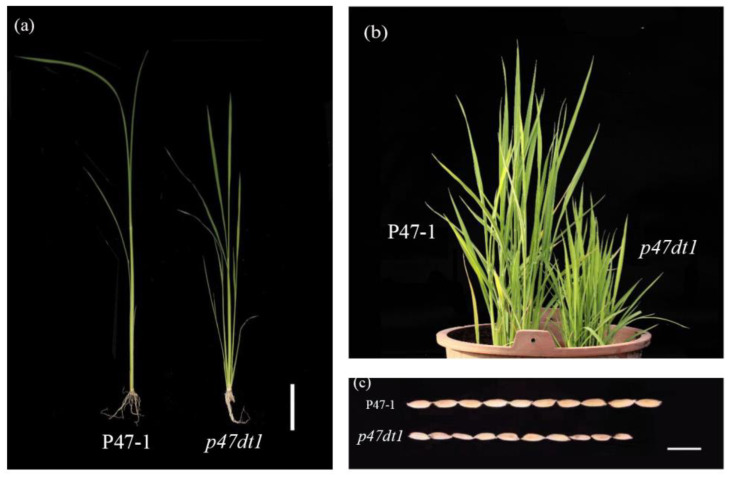
Phenotypes of P47-1 and *p47dt1.* (**a**) Seedling phenotypes of P47-1 and *p47dt1*, with a scale of 2 cm; (**b**) tiller stage phenotypes of P47-1and *p47dt1*, with a scale of 10 cm; (**c**) grain phenotypes of P47-1 and *p47dt1*, with a scale of 1 cm.

This study found a dwarf and multiple tillering mutant *p47dt1,* isolated from high-generation line P47, in the field environment. The tillering number of *p47dt1* was higher than that of P47-1 under outdoor potted planting conditions. Plant height of *p47dt1* was significantly lower than that of P47-1 (Figure 1a,b). The tillers of P47-1 were tall and stout, while those of *p47dt1* were many, short and thin. In addition, there were significant differences between P47-1 and *p47dt1* in grain phenotypes after maturity; P47-1 was larger than *p47dt1* in length, width, and thickness (Figure 1c).

Due to the obvious differences in phenotype, agronomic trait parameters of the mutant and wild type were measured, and it was found that the main agronomic traits of the two showed significant differences in many aspects (Table 1). The average plant height of P47-1was 94.80 cm, and that of *p47dt1* was 55.33 cm, 41.6% lower than that of P47-1. The average tiller number of P47-1 was 16.1, and that of *p47dt1* was 84.5, 5.26 times that of P47-1. The average effective panicle number of P47-1 was 16.3, and that of *p47dt1* was 34.3, 2.1 times that of P47-1. Although the number of effective panicles of *p47dt1* was more than that of P47-1, the number of solid grains, 1000-grain weight and seed setting rate of *p47dt1* were significantly lower than those of P47-1, which indicated that, although the number of tillers of *p47dt1* was significantly increased, most of them were ineffective tillers, and the seed setting rate and 1000-grain weight were very low. This mutation would lead to a decrease in yield.

### 2.2. Dwarfing Multiple Tiller Traits of p47dt1 Were Controlled by a Single Recessive Gene

To identify the genetic characteristics of the mutant, the F_2_ population, which was obtained by backcrossing wild-type P47-1 and mutant *p47dt1* and then self-crossing, was used as the genetic population. One hundred and fifty seeds from the isolated family were sown, and phenotypic observation was carried out. It was found that wild-type phenotypes and dwarf and multiple tillering phenotypes were isolated from F_2_ families, including 113 individuals with the P47-1 phenotype and 37 individuals with the *p47dt1* phenotype. The SPSS Chi-Square test showed that the ratio of wild type to mutant supertype followed the 3:1 separation law (Table 2), indicating that a single recessive gene controlled the dwarfing and multi-tillering traits of the mutant *p47dt1*.

### 2.3. P47DT1 as a Putative Allele of D10

**Figure 2:**
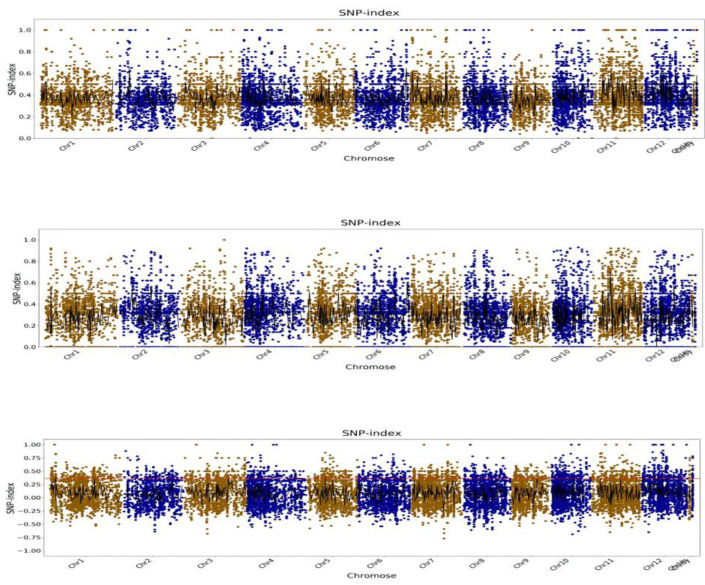
Distribution map of Chromosome SNP-index. The abscissa is the chromosome name, the colored dots represent the calculated SNP-index (or ΔSNP-index) value, and the black line is the fitted SNP-index (or ΔSNP-index) value. The upper picture is the distribution of SNP-index values in the F_2_M pool; the middle picture is the distribution of SNP-index values in the F_2_W pool; the lower picture is the distribution of ΔSNP-index values, in which the red lines represent 99 percent, respectively. Threshold line for digits.

SNP data were filtered, and the SNP index was used for correlation analysis. The 99th percentile of the fitted ΔSNP-index value <0.3 was used as the association threshold to extract genes in the association region (Figure 2). This threshold was chosen to focus on SNPs likely to be associated with the genetic trait being studied, improving the accuracy of candidate region mapping. A total of 62 SNPs were identified after screening SNP sites.

**Figure 3:**
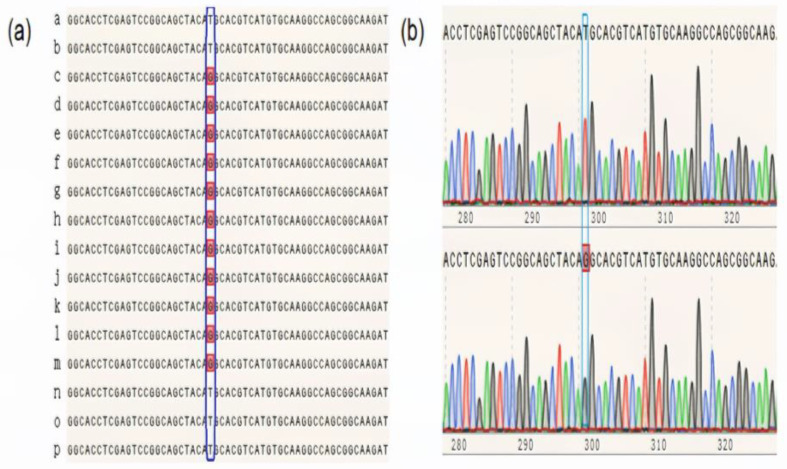
Sequencing analysis of mutation sites in wild-type and mutant and their progeny populations. In figure (**a**), a-b is the mutation site sequence of P47-1; c-d is the mutation site sequence of *p47dt1*; e–m is the mutation site sequence of the *p47dt1* phenotype in the F_2_ population; n–p is the mutation site of the P47-1 phenotype in the F_2_ population Point sequence. (**b**) Shows the mutation site sequence and peak plot of P47-1; the lower figure shows the mutation site sequence and peak plot of *p47dt1*. The blue squares indicate the positions of mutation sites in the sequences, while the red squares highlight the corresponding peaks in the plot that represent these mutations.

Upon comparing marker sites, it was found that, among the possible SNP candidate markers, there was a point mutation (T to G) at position 31,226,439 bp on chromosome 1 within the cloned *D10* gene region, located in an exon (Figure 3). This mutation caused the encoded amino acid to change from methionine (M) to arginine (R). To validate this mutation, PCR amplification and gene sequencing were performed on P47-1, *p47dt1*, and *p47dt1*’s parental mixed DNA samples. The samples were then sent to Shanghai Sangon Biotech Co., Ltd. (Shanghai, China) for gene sequencing.

Sequencing results aligned with the BSA findings, confirming a T-to-G mutation at position 31,226,439 on chromosome 1 in *p47dt1*. To investigate the potential linkage between this mutation and the candidate gene *D10*, we selected wild-type and mutant phenotypes from the F_2_ segregating population of P47-1/*p47dt1* for pooled sequencing analysis (Figure 3). The results indicated that this mutation site also displayed a T-to-G change in the *p47dt1* offspring population, with a strong association to the dwarf and multi-tillering phenotype (Figure 3 and Figure S1, Table S1). Therefore, these results suggest that *P47DT1* is tightly linked to the candidate gene *D10*, making it a putative allele of *D10*.

### 2.4. P47DT1 Changes the Response Pathway of Nitrogen Concentration in Rice

**Figure 4:**
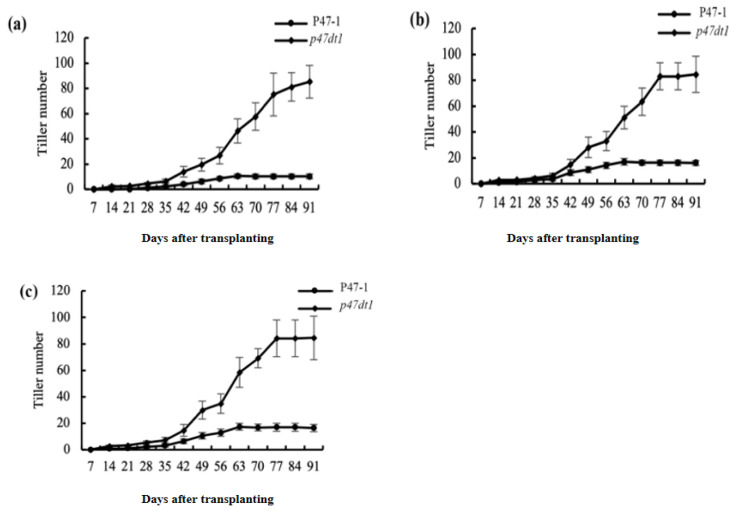
Tiller number and its difference between P47-1 and *p47dt1* under different nitrogen concentrations. (**a**–**c**) Tiller number of P47-1 and *p47dt1* in different periods under low nitrogen (5 g/m^2^), normal nitrogen (10 g/m^2^) and high nitrogen (15 g/m^2^) treatments. The tiller number was measured at different time points (7 to 91 days) after transplanting. Error bars represent the standard error of the mean (SEM) from three biological replicates.

Hormone regulation and nutrient level are the main factors regulating the tillering number of rice plants [4,29,30], so we speculated that *P47DT1* may also change the normal nitrogen response pathway of rice. By treating P47-1 and *p47dt1* with three nitrogen concentrations, it was found that P47-1 increased by an average of 6 tillers from low nitrogen to normal nitrogen with a strong nitrogen response, and increased by an average of 0.33 tillers from normal nitrogen to high nitrogen with a weak nitrogen response. However, there was no difference in the tillering number of *p47dt1* under low nitrogen, normal nitrogen and high nitrogen treatments (Figure 4), and the response to nitrogen was always weak, indicating that the mutation of *P47DT1* changed the normal nitrogen response of rice. In addition, according to the difference in tillering number between P47-1 and *p47dt1* under different nitrogen concentrations, the tillering number of P47-1 was less than that of *p47dt1* with the advance of the growth period, suggesting that *P47DT1* was the key gene controlling the tiller response to nitrogen in rice.

**Figure 5:**
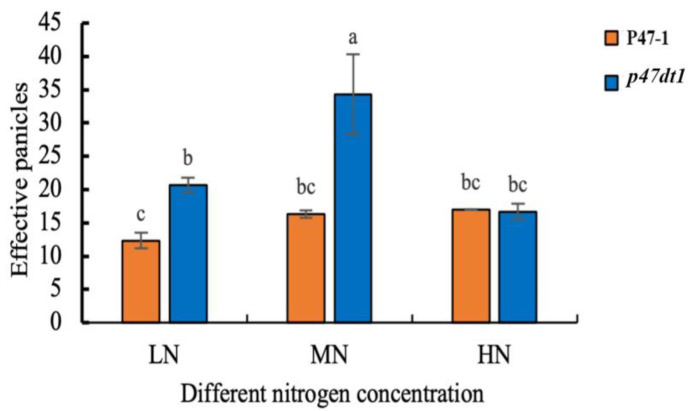
Effective panicles (the panicles that successfully produce grains, excluding sterile or empty panicles) of P47-1 and *p47dt1* under different nitrogen concentrations. Different lowercase letters on the same bar chart indicate significant differences at the 0.05 level. LN represents low nitrogen treatment (5 g/m^2^), MN represents normal nitrogen treatment (10 g/m^2^), and HN represents high nitrogen treatment (15 g/m^2^), as below.

Tillering number determines effective panicle number (the panicles that successfully produce grains, excluding sterile or empty panicles) to some extent, and effective panicle number is the basis of yield formation. Under different nitrogen concentrations, the effective panicle number of P47-1 showed a significant difference under low nitrogen and normal nitrogen treatments but no significant difference under normal nitrogen and high nitrogen treatments, which was consistent with the trend of the tillering number of P47-1 under different nitrogen concentrations. The effective panicle number of *p47dt1* showed significant differences and different trends under different nitrogen concentrations, which increased significantly from low nitrogen to normal nitrogen and decreased significantly from normal nitrogen to high nitrogen (Figure 5). In conclusion, the mutation of *P47DT1* made a significant difference compared to P47-1 in effective panicle number, which also indicated that the mutation affected the normal nitrogen absorption and transformation function of rice.

**Figure 6:**
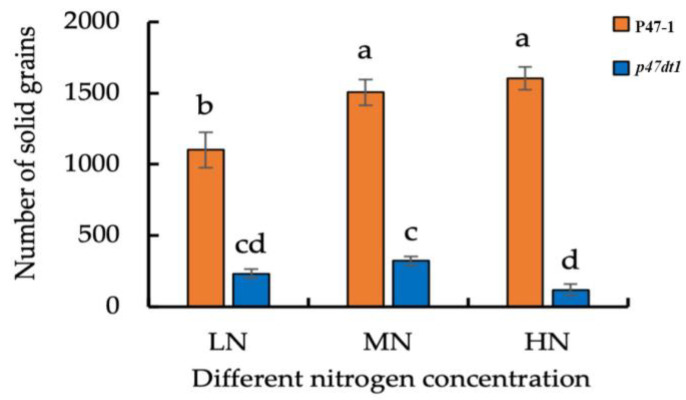
Number of solid grains of P47-1 and *p47dt1* under different nitrogen concentrations. Different lowercase letters on the same bar chart indicate significant differences at the 0.05 level. LN represents low nitrogen treatment (5 g/m^2^), MN represents normal nitrogen treatment (10 g/m^2^), and HN represents high nitrogen treatment (15 g/m^2^).

**Figure 7:**
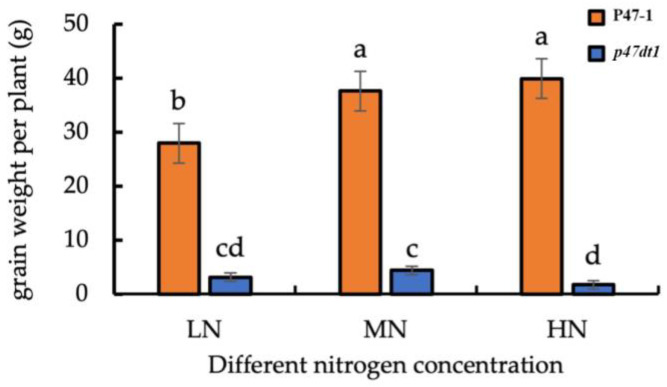
Grain weight per plant for P47-1 and *p47dt1* under different nitrogen concentrations. Different lowercase letters on the same bar chart indicate significant differences at the 0.05 level. LN represents low nitrogen treatment (5 g/m^2^), MN represents normal nitrogen treatment (10 g/m^2^), and HN represents high nitrogen treatment (15 g/m^2^).

In addition, the number of solid grains of P47-1 under normal nitrogen treatment was significantly higher than that under low nitrogen treatment, but there was no significant difference between normal nitrogen treatment and high nitrogen treatment, which was consistent with the change law of effective panicle number under different nitrogen treatments. The number of solid grains of *p47dt1* under normal nitrogen treatment was significantly higher than that under low nitrogen and high nitrogen treatment. Different from P47-1, the number of solid grains of *p47dt1* from normal nitrogen to high nitrogen decreased instead of increasing, and the number of solid grains under high nitrogen treatment was less than that under low nitrogen treatment (Figure 6). As the nitrogen concentration increases, the change in grain weight per plant of wild-type and mutant is similar to the change in the number of solid grains (Figure 7). This result indicated that the mutation of *P47DT1* changed the response mode of rice to exogenous nitrogen concentration.

### 2.5. P47DT1 Alters Nitrogen Allocation Patterns in Rice

**Figure 8:**
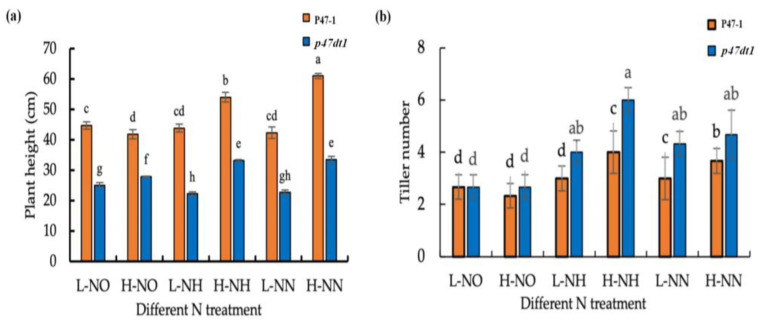
Phenotypic comparison of P47-1and *p47dt1* under hydroponic treatment with different nitrogen conditions. (**a**) Plant height of P47-1 and *p47dt1* under different nitrogen treatments; (**b**) tiller number of P47-1 and *p47dt1* under different nitrogen conditions. The scales in Figures (**a**,**b**) are 15 cm. Different lowercase letters on the same bar chart indicate significant differences at the 0.05 level. L-NO represents 0.2 mM potassium nitrate treatment, H-NO represents 1.5 mM potassium nitrate treatment, L-NH represents 0.2 mM ammonium chloride treatment, H-NH represents 1.5 mM ammonium chloride treatment, L-NN represents 0.2 mM ammonium nitrate treatment, and H-NN represents 1.5 mM ammonium nitrate treatment.

Rice primarily absorbs nitrogen in the forms of nitrate and ammonium, with each form following distinct uptake and assimilation pathways [31,32]. We hypothesized that the *p47dt1* mutation may affect specific pathways, leading to altered nitrogen responses. To investigate these potential pathway changes, P47-1 and *p47dt1* were treated with potassium nitrate, ammonium nitrate, and ammonium chloride. Results showed that, for P47-1, plant height increased significantly with high potassium nitrate concentration, while tiller numbers remained consistent across treatments. In contrast, treatments with ammonium nitrate and ammonium chloride resulted in significantly greater plant height and tiller numbers under high concentrations. In *p47dt1*, tiller numbers showed minimal differences between high and low concentrations across all nitrogen sources but were notably lower with potassium nitrate compared to ammonium nitrate or ammonium chloride (Figure 8). This suggests that both P47-1 and *p47dt1* display insensitivity to nitrate nitrogen, with *p47dt1* exhibiting an even greater insensitivity. Thus, we inferred that *P47DT1* may influence nitrate nitrogen absorption pathways, making the mutant highly insensitive to nitrate nitrogen.

**Figure 9:**
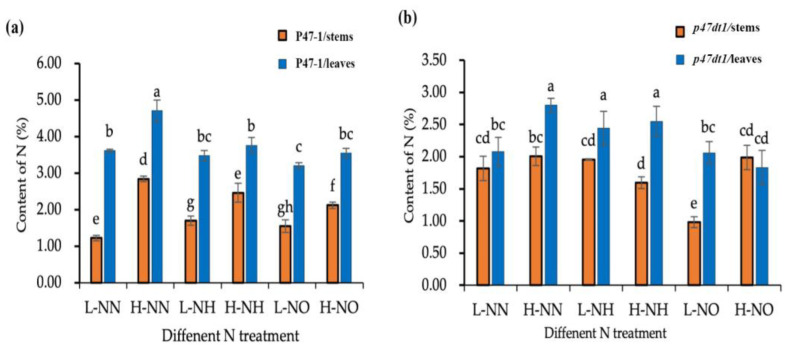
Nitrogen content of P47-1 and *p47dt1* under different nitrogen conditions. (**a**) Comparison of nitrogen content in stems and leaves of wild type P47-1 under different nitrogen conditions; (**b**) comparison of nitrogen contents in stems and leaves of mutant *p47dt1* under different nitrogen conditions. Different lowercase letters on the same bar chart indicate significant differences at the 0.05 level. L-NO represents 0.2 mM potassium nitrate treatment, H-NO represents 1.5 mM potassium nitrate treatment, L-NH represents 0.2 mM ammonium chloride treatment, H-NH represents 1.5 mM ammonium chloride treatment, L-NN represents 0.2 mM ammonium nitrate treatment, and H-NN represents 1.5 mM ammonium nitrate treatment.

To further assess if *P47DT1* mutation affects nitrogen allocation within the plant, both P47-1 and *p47dt1* were treated under six nitrogen conditions: 0.2 mM potassium nitrate, 1.5 mM potassium nitrate, 0.2 mM ammonium chloride, 1.5 mM ammonium chloride, 0.2 mM ammonium nitrate and 1.5 mM ammonium nitrate. Nitrogen content in P47-1 leaves was significantly higher than in stems across all treatments, while *p47dt1* showed higher nitrogen content in leaves under ammonium-based treatments but had elevated stem nitrogen under low potassium nitrate conditions (Figure 9). These findings indicate that *P47DT1* mutation affects nitrogen allocation between leaves and stems through nitrate nitrogen pathways.

### 2.6. Potential Role of TCP19 in Regulating D10 Expression

**Figure 10:**
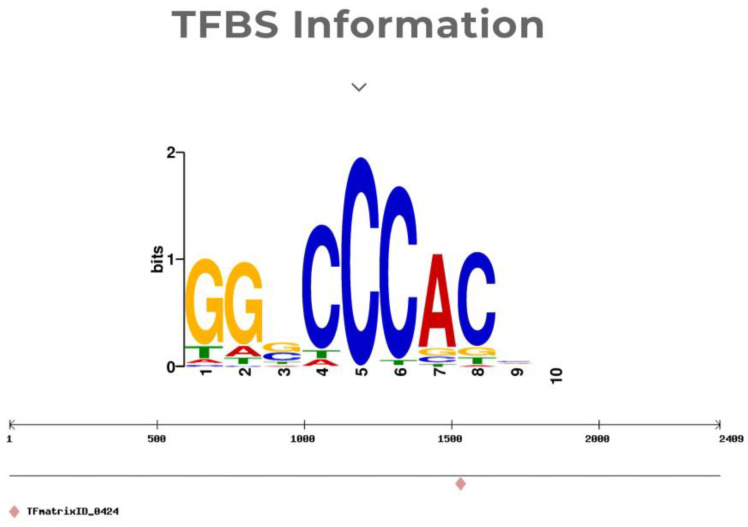
The target sequence of TCP19 and the putative binding position on the *D10* promoter. Here TFBS represents Transcription Factor Binding Site. The TFBS Information visualizes the motif recognized by a particular transcription factor, identified by a matrix (TFmatrixID_0424), with the sequence “GGCCCAC”. This shows the binding motif that the transcription factor is likely to interact with in the genome. Blue highlights indicate the regions of the promoter where TCP19 is predicted to bind, while red highlights indicate regions where the transcription factor may have a stronger binding affinity. The figure also illustrates the position of the binding motif in the genome.

To analyze how the *p47dt1* mutation might influence the nitrogen absorption pathway, the transcription factor prediction of the promoter of the *D10* gene was performed by the bioinformatics method. As *D10* is completely linked to *P47DT1*, the 2.4 kb promoter sequence upstream of the start codon of the *D10* gene was predicted, revealing that the promoter of this gene contains a large number of elements related to plant hormone response. *D10* was involved in the biosynthesis of strigolactone derivative SL and should also be involved in biological processes regulated by plant hormones. According to previous experiments, *D10* was also found to participate in nitrogen response [23]. Therefore, transcription factors related to nitrogen and plant hormones were selected as screening conditions for prediction results analysis (Figure 10). A TCP family transcription factor TCP20 (*Arabidopsis thaliana*) was found at Matrix ID 0424. It was speculated that this position might be combined with other TCP family transcription factors in rice, among which the transcription expression of *Os06g0226700 (TCP19)* was negatively regulated by external nitrogen level. As a transcription factor, TCP19 can promote the expression of gene *DLT* and thus realize the regulation of rice tiller development. Both *D10* and *DLT* regulate tillering number in rice through the hormone pathway, and both were related to nitrogen. Therefore, it was speculated that *D10* may be involved in another related pathway in the OsTCP19-mediated nitrogen regulation network.

**Figure 11:**
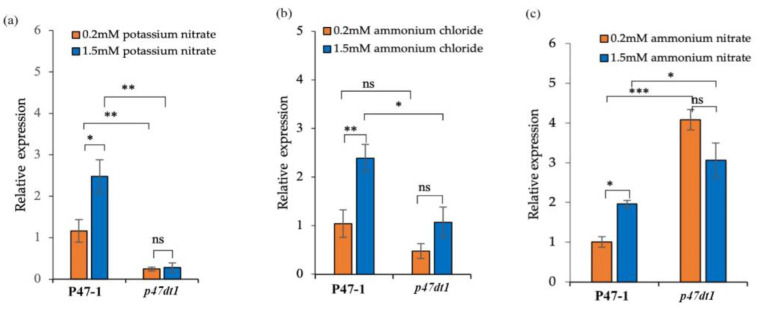
Relative expressions of *D10* in P47-1 and *p47dt1* under different nitrogen conditions. (**a**) Relative expressions of *D10* of P47-1 and *p47dt1* treated with potassium nitrate of 0.2 mM and potassium nitrate of 1.5 mM; (**b**) relative expressions of *D10* of P47-1 and *p47dt1* treated with 0.2 mM ammonium chloride and 1.5 mM ammonium chloride; (**c**) relative expressions of *D10* of P47-1 and *p47dt1* treated with 0.2 mM ammonium nitrate and 1.5 mM ammonium nitrate. Data are mean ± standard deviation (*n* = 10); student’s *t*-test, * means significant at 0.05 level; ** means significant at 0.01 level; *** means extremely significant at 0.001 level; ns means no significant difference.

The expression of *D10* in the wild-type plant P47-1 significantly increased with higher nitrogen concentrations across different nitrogen forms (Figure 11a–c). This is consistent with the feedback mechanism of strigolactone biosynthesis, where reduced strigolactone synthesis caused by nitrogen promotes *D10* expression [33]. However, in the mutant *p47dt1*, this trend was not observed, suggesting that the mutation disrupted the strigolactone synthesis pathway, leading to altered feedback and elevated *D10* expression (Figure 11c).

**Figure 12:**
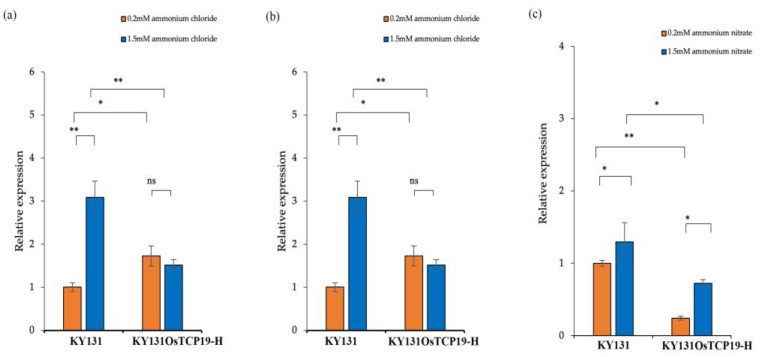
Relative expressions of *D10* in KY131 and KY131^OsTCP19-H^ under different nitrogen conditions. (**a**) Relative expressions of *D10* of KY131 and KY131^OsTCP19-H^ treated with potassium nitrate of 0.2 mM and 1.5 mM; (**b**) relative expressions of *D10* of KY131 and KY131^OsTCP19-H^ treated with 0.2 mM ammonium chloride and 1.5 mM ammonium chloride; (**c**) relative expressions of *D10* of KY131 and KY131^OsTCP19-H^ treated with 0.2 mM ammonium nitrate and 1.5 mM ammonium nitrate. Data are mean ± standard deviation *(n* = 10); student’s *t*-test, * means significant at 0.05 level; ** means significant at 0.01 level; ns means no significant difference.

To investigate the relationship between TCP19 and *D10*, we used nitrogen-efficient KY131*^OsTCP19^*^-H^ and nitrogen-inefficient KY131. These materials were selected for their contrasting nitrogen efficiency and differences in *TCP19* expression levels. Notably, *TCP19* expression was significantly lower in KY131*^OsTCP19^*^-H^ compared to KY131 under the same nitrogen conditions (Figure 12c) [23]. Additionally, *D10* expression in KY131 and KY131*^OsTCP19^*^-H^ showed a conserved response to increasing nitrogen concentration, aligning with patterns observed in P47-1. This trend suggests a possible involvement of TCP19 in the regulation of *D10* under nitrogen conditions. However, these findings demonstrate an association and are not definitive evidence of a direct regulatory mechanism.

**Figure 13:**
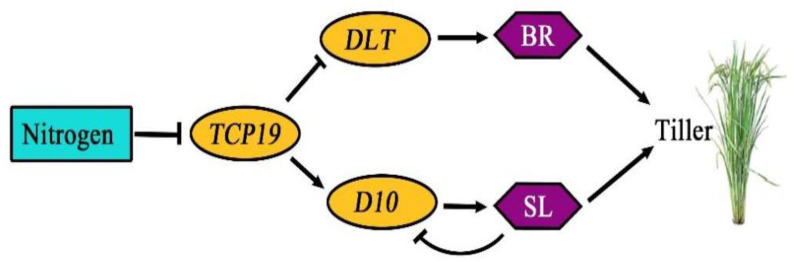
A proposed model for *D10* in TCP19-mediated tiller development response to nitrogen. The diagram shows the nitrogen response pathway influencing rice tillering. TCP19 represents a transcription factor that regulates tiller formation in response to nitrogen. DLT (Dwarf and Low-Tillering transcription factor involved in brassinosteroid (BR) signaling, essential for regulating cell growth and tillering), SL (strigolactones), (BR) brassinosteroid.

Previous research has shown that nitrogen negatively regulates TCP19 primarily via the nitrate pathway [23]. As TCP19 can inhibit the expression of the tiller-promoting gene *DLT* through the brassinosteroid pathway, it is speculated that TCP19 might also influence nitrogen responses involving *D10* and strigolactones. However, the exact mechanisms linking TCP19 to *D10* remain speculative and require further validation (Figure 13).

In conclusion, while the results suggest that TCP19 may influence *D10* expression and nitrogen response in rice, these findings remain speculative. Further experimental studies are needed to confirm the regulatory pathways and their implications for tiller development.

## 3. Discussion

### 3.1. P47DT1 Mutation Affects Nitrogen Absorption Pathways

Many agronomic characteristics, such as plant height, tiller number, effective panicle number, and grain number, were significantly different between P47-1 and *p47dt1* after potted soil culture with low nitrogen, normal nitrogen, and high nitrogen. P47-1 showed a significant tiller response to nitrogen from low nitrogen to normal nitrogen but no significant change in tiller response to nitrogen from normal nitrogen to high nitrogen. This verifies the conclusion of previous studies that rice’s nitrogen absorption has a threshold and, when the nitrogen absorbed by rice meets its own growth and development needs, it will not continue to absorb and utilize soil nitrogen [34]. Therefore, in the actual process of rice production, we should not blindly think that more nitrogen fertilizer can increase the yield. Overuse of nitrogen can result in groundwater contamination, higher production costs, decreased crop yields, and overall environmental pollution [35].

However, *p47dt1* showed almost the same tiller response to nitrogen under low nitrogen, normal nitrogen, and high nitrogen conditions, indicating that the mutation also affected the normal nitrogen response of rice. Although the *p47dt1* mutant exhibited a higher tiller number under high nitrogen concentration, the number of effective panicles did not increase correspondingly. This suggests that the mutation impacts the plant’s ability to transform the increased tillering into productive panicles, likely due to reduced nitrogen absorption efficiency and transformation into grains.

When *p47dt1* was treated with nitrate nitrogen, its phenotype changes at high and low concentrations were different from those under other nitrogen conditions. It was preliminarily concluded that *P47DT1*/*D10* might be related to the nitrate nitrogen absorption pathway, and it was extremely insensitive to nitrate nitrogen after mutation. The *p47dt1* mutation appears to affect nitrogen use efficiency rather than total nitrogen uptake, as evidenced by the lower nitrogen content in the mutant tissues (Figure 9). Although the mutant *p47dt1* produces more tillers, these tillers are less efficient at utilizing nitrogen to form effective panicles, resulting in fewer grains and lower overall yield.

Additionally, although the *p47dt1* mutant forms more tillers, the number of solid grains and the grain weight per plant are significantly lower than in the wild type (Figure 7 and Figure 8). This may be attributed to inefficient nutrient partitioning and nitrogen use in the mutant, where increased tiller production does not result in higher yield. The reduced ability to absorb and effectively utilize nitrogen for grain development may explain the lower solid grain numbers and grain weight in the mutant.

Thus, the *p47dt1* mutation affects nitrogen use efficiency, leading to lower grain yield despite increased tillering. This reduced efficiency is reflected in lower nitrogen content in the plant tissues (Figure 9) and fewer solid grains, as shown in (Figure 6 and Figure 7). This demonstrates that the mutation altered the normal nitrogen uptake and distribution rules of rice, impacting the overall agronomic performance.

### 3.2. D10’s Possible Role in the TCP19-Mediated Nitrogen Response Pathway

There is a conserved feedback inhibition mechanism between the strigolactone synthesis gene *D10* and strigolactone (SL). Under nitrogen limitation, enhanced SL biosynthesis suppresses *D10* expression due to negative feedback regulation. Conversely, when nitrogen is abundant, SL synthesis decreases, relieving feedback inhibition and increasing *D10* expression. In the mutant *p47dt1*, the disruption of the SL synthesis pathway appears to alter this feedback mechanism. As a result, *D10* expression remains elevated, likely due to enhanced feedback effects, with expression levels significantly higher than in the wild-type plant P47-1.

To further explore potential regulatory pathways involving *D10*, transcription factors related to nitrogen and plant hormone signaling were predicted based on the *D10* promoter sequence. Among these, TCP19 was identified as a candidate transcription factor potentially influencing nitrogen responses in rice. Previous studies have suggested that TCP19 might regulate the expression of tiller-promoting genes through pathways such as brassinosteroids, and its expression is responsive to nitrogen. However, the specific interaction between TCP19 and *D10* remains speculative.

The relevance of KY131 and KY131*^OsTCP19^*^-H^ to the present study lies in their contrasting nitrogen efficiency, which provides a comparative framework for investigating the role of TCP19 and *D10* in nitrogen response and tillering regulation. KY131 is nitrogen-inefficient, while KY131*^OsTCP19^*^-H^ is nitrogen-efficient, presumably due to alterations in *TCP19* expression levels. This distinction allows researchers to explore the relationship between nitrogen-responsive transcription factors and their downstream effects on genes like *D10*, which are involved in the nitrogen absorption and tillering pathways. Using these materials, it was observed that *TCP19* expression was significantly lower in KY131*^OsTCP19^*^-H^ under the same nitrogen conditions. This trend was consistent across various nitrogen treatments, aligning with observations in P47-1. These findings suggest that TCP19 may influence *D10* expression under nitrogen-regulated conditions. However, it should be noted that the observed association between *TCP19* and *D10* expression patterns is not definitive evidence of a direct regulatory relationship. Instead, these findings highlight a possible involvement of TCP19 in the nitrogen response pathway in which *D10* might participate.

Although *TCP19* and *D10* exhibit significant expression differences between nitrogen-efficient and nitrogen-inefficient materials, the exact regulatory mechanism remains unclear. Further studies are needed to determine whether TCP19 directly regulates *D10* or if the observed patterns result from broader transcriptional networks responding to nitrogen. Therefore, while our results suggest that *P47DT1/D10* modulates nitrogen response and tiller development, this regulation may occur indirectly or through additional pathways influenced by TCP19.

In conclusion, *D10* is likely to participate in a TCP19-mediated nitrogen response pathway, potentially affecting tiller responses under varying nitrogen conditions. However, this conclusion is speculative, and additional studies are required to validate these interactions and their implications for rice tillering regulation.

## 4. Materials and Methods

### 4.1. Experimental Materials

The experiment was conducted in the pot farm of Northeast Agricultural University (126.72° E, 45.75° N) in 2021. The mutant *p47dt1* was used as the test material and the wild-typeP47-1 was used as the control material for phenotypic analysis, genetic analysis, gene mapping and nitrogen response analysis. KY131^OsTCP19-H^ was used as test material and KY131 was used as control material for nitrogen response analysis The lines KY131 and KY131^OsTCP19-H^ were provided by Professor Chu Chengcai from South China Agricultural University and are used for nitrogen response analysis, and the natural mutant *p47dt1* of the high-generation rice material P47-1 was discovered in a field environment.

### 4.2. Population Construction and Separation Statistics

F_2_ population used was derived from a cross between the P47-1 wild-type rice and the *p47dt1* mutant, which exhibits a dwarf and multi-tillering phenotype. After obtaining F_1_ seeds from the cross, these seeds were self-pollinated to generate the F_2_ population.

All the F_2_ generation seeds harvested underwent broken dormancy for 16 h with 0.6% dilute HNO_3_, then washed three times with clean water to wash away the dilute nitric acid and replaced with distilled water to promote germination in the dark at 37 °C. After the seeds’ dewing, they were sown in the rice nutrient soil for seedling cultivation (Culture temperature: 30 °C in the daytime, 22 °C at night; photoperiod: 12 h during the day, 12 h at night; ambient humidity: relative humidity (RH) is about 80%; light intensity: 30,000 Lux). At the tillering stage, the number of individuals with P47-1 phenotype and *p47dt1* phenotype isolated from F_2_ generation was counted based on phenotypic differences.

### 4.3. Gene Mapping

The CTAB method was used to extract DNA from 30 independent P47-1 phenotypic individuals, 30 independent *p47dt1* phenotypic individuals, and the parental line (P47-1). For each genotype, a total of 30 DNA samples were collected. After extraction, the DNA samples were pooled by genotype, resulting in three bulk DNA samples: (1) a bulk sample consisting of DNA from 30 P47-1 phenotypic individuals; (2) a bulk sample consisting of DNA from 30 *p47dt1* phenotypic individuals; (3) a bulk sample consisting of DNA from the P47-1 parental line.

These three pooled DNA samples were then sent to Frasergen Bioinformatics Co., Ltd. (Wuhan, China) for Bulked Segregant Analysis (BSA) mixed sequencing analysis. The test process includes sample testing, library preparation, library quality testing, and computer sequencing. In order to ensure the quality of information analysis, clean reads were obtained by fine filtering of raw reads, and subsequent analysis was conducted based on clean reads. Specific filtration standards are (1) SNP loci with multiple genotypes, unidentified SNP loci; (2) SNP loci with support of less than 4; (3) SNP loci of mutant progeny with mixed pool genotype consistent with wild type parent genotype or homozygous loci with genotypic consistency among offspring mixed pools; (4) wild type parents heterozygous loci; (5) the SNP-index of the mixed pool genotypes of the mutant and wild offspring was less than 0.3.

The SNP-index method was used to select the candidate regions related to the target traits and their mutation locations and mutation types. SNP-index difference for two offspring pools with extreme traits (ΔSNP index) were used to obtain the ΔSNP-index distribution. Candidate regions were screened within a 0.01 confidence interval. SNP markers and InDel markers were screened. After screening, primers were designed according to the location of candidate sites, and PCR amplification was performed. The amplified products were sent to Shanghai Sangong Biological Co., Ltd. (Shanghai, China) for sequencing.

### 4.4. Pot Test Scheme

Three gradient fertilizer treatments were set, respectively: high nitrogen (50% higher than the normal amount, HN): 15 g/m^2^, normal nitrogen (control, MN): 10 g/m^2^, low nitrogen (50% lower than the normal amount, LN): 5 g/m^2^; 10 replicates were set for each treatment, and all treatments of phosphate fertilizer, potassium fertilizer and organic fertilizer were consistent. Nitrogen fertilizer is urea (N ≥ 46%), phosphate fertilizer is granular heavy superphosphate calcium (P_2_O_5_ ≥ 46%), and potassium fertilizer is granular potassium chloride (K_2_O ≥ 60%). According to the surface area of the basin, to convert the nitrogen, phosphorus, and potassium fertilizer application amount of each basin, the ratio of nitrogen, phosphorus, and potassium was 1:0.5:0.8. The fertilization method uses all phosphate fertilizer as base fertilizer; 50% of potassium fertilizer is used as base fertilizer and 50% as ear fertilizer; 50% of Nitrogen fertilizer is used as base fertilizer, 30% as tillering fertilizer, and 20% as ear fertilizer. The application was divided into three stages: before transplanting (base fertilizer application in mid-May), at the end of rejuvenation (tiller fertilizer application in late May), and at the booting stage (ear fertilizer application in early July).

### 4.5. Hydroponics Test Scheme

Because the content of (NH_4_)_6_Mo_7_O_24_·4H_2_O in the formula of the International Rice Research Institute [31] is very low, it is only necessary to replace and adjust the amount and type of NH_4_NO_3_ in the mother liquor for nitrogen-treated medium with different forms and concentrations. For specific adjustments, please refer to (Table 3). Seeds of P47-1, *p47dt1*, KY131, and KY131^OsTCP19-H^ were sowed on the mesh plates, and the mesh plates were placed in Syngenta transparent boxes without any treated tap water. Replace the water every 3 days until the nutrition of the seeds is exhausted (pinch the seeds to show a withered state), then use different nitrogen treatments of culture medium for culture, each treatment consisting of 3 repeats every 3 days to replace the culture medium. On the 21st day of culture in the growth chamber, the plant height and tiller number of P47-1 and *p47dt1* were measured, and materials consistent with the mean value were selected for photo recording. On the 33rd day, roots of four kinds of materials were taken under different treatment conditions for RNA extraction, reverse transcription, fluorescence quantification, and other tests.

### 4.6. Nitrogen Content Determination

Leaves and stems of P47-1 and *p47dt1* treated with different nitrogen conditions were dried, ground into powder, and boiled. The total nitrogen content in leaves and stems was determined by the Kjeldahl nitrogen determination method [36].

### 4.7. Transcription Factor Prediction

The *D10* promoter gene sequence was obtained from the NCBI website (https://www.ncbi.nlm.nih.gov), and then this sequence was entered into the promoter analysis function dialogue box of PLANTPAN online website (https://plantpan.itps.ncku.edu.tw), and rice was selected as the analyzed species to perform transcription factor prediction analysis of promoter binding.

### 4.8. Determination of Gene Expression

RNA from the roots of P47-1, *p47dt1*, KY131, and KY131*^OsTCP19^*^-H^, treated with different nitrogen conditions, was extracted using the Trizol method. The sequence of gene *D10* was obtained at NCBI, and the accuracy of the sequence was tested by BLAST. The online website Primer 3 (https://primer3.ut.ee) was used to screen the CDS region sequence of *D10* and, after designing the primer length and product size (Table 4), appropriate *D10* and *TCP19* primers were selected and synthesized by Beijing Ruiboxingke Biological Co., Ltd. (Beijing, China) [20]. RNA reverse transcription was performed using Novozan HiScript III 1st Strand cDNA Synthesis Kit (+gDNA wiper), and then quantitative qRT–PCR was performed on cDNA of rice root samples. *D10* and *TCP19* gene expression data were analyzed and processed by the 2^−ΔΔCt^ method. the QUBI gene was used as the reference gene for normalizing the expression levels of the target genes.

### 4.9. Data Analysis

Analysis of variance (ANOVA), followed by post hoc least significant difference (LSD), was carried out using SPSS software (IBM Corp., Armonk, NY, USA). LSD at 5% (*p* ≤ 0.05) probability levels was employed for pairwise means comparison analysis. A Two-Way ANOVA was performed to analyze the effects of two independent variables: nitrogen concentration (levels: LN, MN, HN for Figure 5, Figure 6 and Figure 7; and levels: L-NO, H-NO, L-NH, H-NH, L-NN, H-NN for Figure 9 and Figure 10) and variety (wild-type P47-1 and mutant *p47dt1*) on the dependent variables. This approach allowed precise identification of treatment effects and interactions under controlled conditions [37].

### 4.10. Graphical Software

The experimental data were mainly processed using Excel 2007, SPSS 22.0, Graph prism 8, Photoshop CS5, and other software for data significance analysis and chart making.

## 5. Conclusions

The dwarf and multiple tillering traits of the *p47dt1* mutant are controlled by a single recessive gene, and gene mapping suggests that *P47DT1* is a putative allele of *D10*. The mutation in *P47DT1* appears to alter the nitrogen response pathway and the general pattern of nitrogen allocation in rice, potentially involving the nitrate nitrogen pathway. This mutation may also interact with pathways regulating tillering in rice, including those mediated by TCP19. While our findings suggest a possible positive regulation of *P47DT1*/*D10* by TCP19, further experimental validation is required to clarify the underlying mechanisms.

This study also demonstrated that more nitrogen does not always lead to better yield, highlighting the importance of efficient nitrogen management. The mutation increases tillering but does not enhance effective grain production, suggesting that future breeding efforts should focus on achieving a balance between tiller number and grain formation for improved productivity. Furthermore, insights into *P47DT1* and TCP19 can guide breeding programs to develop rice varieties with better nitrogen use efficiency, contributing to higher yields with fewer resources. This research underscores the importance of nitrogen-efficient crop varieties for sustainable agriculture, reducing fertilizer use while maintaining high productivity.

## Figures and Tables

**Table 1:** Comparison of agronomic traits between P47-1 and *p47dt1*.

Agronomic Traits	P47-1	*p47dt1*
Plant height (cm)	94.80 ± 2.73	55.33 ± 3.79 **
Number of tillers	16.1 ± 1.9	84.5 ± 14.0 ***
Panicle length (cm)	16.33 ± 0.57	10.73 ± 0.21 **
Effective panicles	16.3 ± 0.5	34.3 ± 4.9 **
Total grains per plant	1884.33 ± 116.42	1219.66 ± 132.34 **
Number of solid grains	1505.33 ± 72.96	369.33 ± 65.49 ***
1000-grain-weight (g)	25.03 ± 0.12	13.53 ± 0.24 **
Seed setting rate (%)	80.00 ± 4.54	30.66 ± 9.56 ***

Data are mean ± standard deviation (*n* = 10); student’s *t*-test, ** means significant at 0.01 level; *** means extremely significant at 0.001 level.

**Table 2:** Genetic analysis of tiller trait in the F_2_ population.

Population	Population Size	The Phenotype of P47-1	The Phenotype of *p47dt1*	Value of Expectation	χ^2^	χ^2^_(0.05)_
F_2_	150	113	37	3:1	0.009	3.84

**Table 3:** Adjustment table of nitrogen nutrient mother solution.

Stock-2 Drugs	0.2 (mol/L)	1.5 (mol/L)
NH_4_Cl (53.49 g/mol)	10.69 (g/L)	80.23 (g/L)
KNO_3_ (101.1 g/mol)	20.22 (g/L)	151.65 (g/L)
NH_4_NO_3_ (80 g/mol)	16 (g/L)	120 (g/L)

**Table 4:** qRT–PCR primer information.

Gene Name	Accession No.	Forward-Primer (5′-3′)	Reverse-Primer (5′-3′)
*D10*	LOC_Os01g54270	GGAAGAGTGTACGGCAGGAG	GTAGTCGCCGAGGTTCCATA
*TCP19*	LOC_Os06g12230	GACAGTGTACCGTGGCGT	CGCCGGGAAGTTCATGAAAT
*QUBI*	LOC_Os03g13170	GCTCCGTGGCGGTATCAT	CGGCAGTTGACAGCCCTAG

## Data Availability

The datasets presented in this study can be found in online repositories. The names of the repository/repositories and accession number(s) can be found below: NGDC-GSA database under the BioProject no. PRJCA031448 and accession no. CRA019863 for the BSA-seq data.

## Supplementary Materials

The following supporting information can be downloaded at: https://www.mdpi.com/article/10.3390/plants13233349/s1, Table S1: Primer information for gene identification and Figure S1: Mutation positions of the *d10* mutant versus the *p47dt1* mutant.
