# Supplementary material for: Putative Allele of D10 Gene Alters Rice Tiller Response to Nitrogen
Source: Plants (Basel). 2024 Nov 29;13(23):3349. doi: 10.3390/plants13233349 (PMC11644428; doi:10.3390/plants13233349)
Supplement: Supplementary file 1 [file plants-13-03349-s001.zip › Table S1.pdf]

Supplementary Table

Table S1. Primer information for gene identification

Gene	Forward-primer (5′-3′)	Reverse-primer (5′-3′)	Product size(bp)
D10	TCCAGTACACGGACAAGCTG	ATACGTGCATGCGTAGCGTA	723
