# Supplementary figures and images for: Putative Allele of D10 Gene Alters Rice Tiller Response to Nitrogen
Source: Plants (Basel). 2024 Nov 29;13(23):3349. doi: 10.3390/plants13233349 (PMC11644428; doi:10.3390/plants13233349)

Supplementary Figure

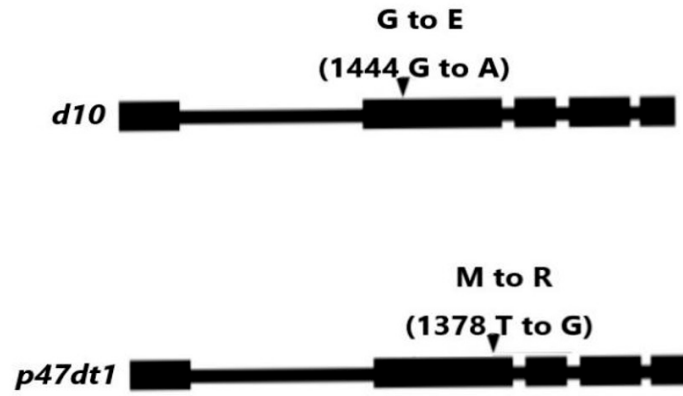

Figure S1: Mutation positions of the *d10* mutant versus the *p47dt1* mutant.

Supplement: Supplementary file 1 [file plants-13-03349-s001.zip › Figure S1.pdf]
